# Ocular Behcet’s disease is associated with aberrant methylation of interferon regulatory factor 8 (IRF8) in monocyte-derived dendritic cells

**DOI:** 10.18632/oncotarget.17235

**Published:** 2017-04-19

**Authors:** Yiguo Qiu, Yunyun Zhu, Hongsong Yu, Shenglan Yi, Wencheng Su, Qingfeng Cao, Gangxiang Yuan, Aize Kijlstra, Peizeng Yang

**Affiliations:** ^1^ The First Affiliated Hospital of Chongqing Medical University, Chongqing Key Laboratory of Ophthalmology, Chongqing Eye Institute, Chongqing, China; ^2^ University Eye Clinic Maastricht, Maastricht, The Netherlands

**Keywords:** DNA methylation, interferon regulatory factor 8, Behcet's disease, dendritic cells

## Abstract

Aberrant methylation of interferon regulatory factor 8 (IRF8) has been noted in various tumors. IRF8 has also been reported to be involved in many autoimmune diseases, including Behcet's disease (BD). However, the methylation status of IRF8 in BD has not been reported. To address this issue, we investigated whether the degree of methylation of IRF8 in dendritic cells (DCs) plays a role in the development of BD. We found a lower mRNA expression and a higher methylation level of IRF8 in active ocular BD patients as compared to normal subjects and inactive patients. Treatment with a demethylation agent, 5-Aza-2’-deoxycytidine (DAC) resulted in an increase of mRNA expression and a reduction of the IRF8 methylation level. It also down-regulated the expression of the co-stimulatory molecules CD86, CD80, CD40, and reduced the production of IL-6, IL-1β, IL-23 and IL-12. An inhibition of Th1/Th17 responses was observed as evidenced by a decreased production of IFN-γ, IL-17, and a reduction of IFN-γ/IL-17- producing CD4^+^ T cells following treatment with DAC. This study shows that active ocular BD patients have an aberrant IRF8 methylation status. These findings suggest that epigenetic control of IRF8 expression may offer a future target in the treatment of ocular BD.

## INTRODUCTION

Behcet's disease (BD) is a systemic autoimmune-mediated inflammatory disease. The intraocular inflammation (uveitis) which often occurs in these patients is a serious sight-threatening clinical entity that is often accompanied by recurrent episodes of oral ulceration, genital ulceration and skin lesions [1]. The pathogenesis of BD remains unclear, but environmental factors, microbial triggers, genetic susceptibility and immunological aberrations have been implicated [2]. Numerous studies have demonstrated that the pathogenesis of BD is closely related to an enhanced T helper (Th) type 1 and Th17 cell immune response [3–5].

As potent antigen presenting cells (APCs), dendritic cells (DCs) are important in inducing primary immune responses, and initiating adaptive immune responses [6]. The co-stimulatory molecules of DCs facilitate the activation of naïve T cells and thus initiate a primary immune response. Moreover, the differentiation of different effector T cells from naïve T cells relies critically upon the particular cytokine environment created by DCs during autoimmune pathology [7, 8]. Notably, various autoimmune inflammatory diseases, such as uveitis, are perceived as being caused by an aberrant immune response in which DCs promote the activation of different subsets of Th cells [6]. Previous studies including work from our team demonstrated that excessive Th1/Th17 responses in ocular BD patients could be inhibited by regulating DC function via immunomodulatory treatments [9–11].

Various genes have been implicated to be involved in BD, including interferon regulatory factor (IRF) 8 [12, 13], which belongs to the IRF family [14]. IRF8 not only regulates the immune response, but it also modulates multiple important pathophysiologic processes, such as cell growth, differentiation and oncogenesis [15]. Additionally, IRF8 plays an important role in regulating the development and function of a variety of immune cells [16, 17]. It also plays a vital role in the development and activation of DCs [18, 19]. It has been demonstrated that a specific remodeling of the chromatin structure at the IRF8 gene is necessary when initiating differentiation of DCs [20]. Moreover, accumulating evidence demonstrates that single nucleotide polymorphisms (SNPs) of IRF8 are associated with several autoimmune diseases, including BD [12, 21, 22]. The aforementioned studies suggest that IRF8 is related to various autoimmune diseases by modulating DCs. A previous study shows that IRF8 is not expressed in naïve T cells until they are stimulated by antigens or following TCR activation, suggesting that IRF8 may be required in the process of regulating genes that are involved in T cell differentiation and/or effector functions [23]. In fact, IRF8 can inhibit Th17 cell differentiation through its interaction with the Th17 master transcription factor, ROR-γt [24]. Moreover, IRF8 is able to regulate innate immune responses and can influence the differentiation of Th cells by modulating transcription of cytokine genes in APCs [25, 26].

Besides a role for SNPs in the function of a gene like IRF8, other mechanisms such as epigenetic regulation may also be involved. Epigenetic regulation mainly includes DNA methylation, histone modification and RNA interference [27]. DNA methylation, which is characterized by adding a methyl group from S-adenosylmethionine to the fifth carbon of cytosine rings within CpG dinucleotides [28], has been increasingly recognized to be essential in the immune response, especially in the differentiation and function of Th cell subsets [29]. Hypomethylation of DNA can activate transcription of genes, while hypermethylation often results in transcriptional gene repression [29].

DNA methylation often occurs in the CpG islands (the guanine and cytosine-rich gene regions) in the promoter region which subsequently affects the transcription of associated genes. A variety of studies have revealed that aberrant methylation changes of the IRF8 promoter are associated with the development of various human tumors [30–32]. Moreover, the changes in DNA methylation have been shown to be important in a number of immune-mediated diseases [33–35]. Whether an aberrant methylation of IRF8 is present in BD patients has not yet been reported and was therefore the purpose of the study described here. Considering the important role of DCs in BD, we focused our study on this population of cells. To explain the effects of IRF8 methylation on the pathogenesis of BD, *in vitro* experiments were performed to evaluate the demethylation effect of IRF8 on DC function. Our findings suggest that hypermethylation of IRF8 is associated with ocular BD. *In vitro* demethylation treatment was shown to be able to regulate the function of DCs and to inhibit Th1/Th17 responses, offering an explanation for the effect of IRF8 methylation in the development of BD.

## RESULTS

### A reduced mRNA expression of IRF8 and a hypermethylation status of IRF8 promoter was observed in DCs from active ocular BD patients as compared to normal controls or inactive BD patients

**Figure 1:**
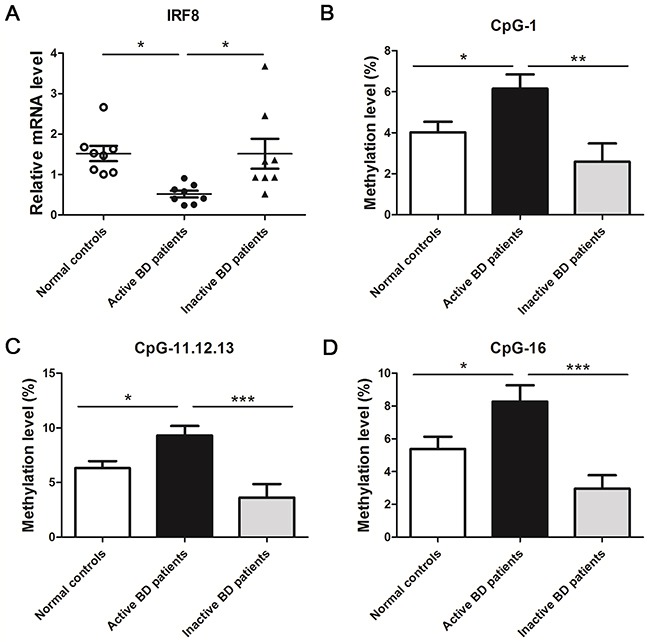
A decreased mRNA level and an increased methylation level of IRF8 in DCs obtained from active BD patients was noted compared with normal controls and inactive BD patients **(A)** DCs from active BD patients, inactive BD patients and normal controls were used to detect the mRNA expression of IRF8 with real-time PCR (normal controls: n = 8; active BD patients: n=8, inactive BD patients: n=8,*p<0.05). Methylation levels of the CpG sites between -441 and -225 from the TSS of the first exon were detected with a MALDI-TOF mass spectrometer in the DCs of active BD patients, inactive BD patients and normal controls. The methylation levels of CpG_1 **(B)**, CpG_11.12.13 **(C)** and CpG_16 **(D)** in the group of active BD patients was significantly higher than that observed in the group of normal controls and inactive patients (normal controls: n = 30; active BD patients: n=20, inactive BD patients: n=15,*p<0.05, **p<0.01, ***p<0.001). The data are shown as mean ± SEM. One way-ANOVA, followed by Bonferroni correction was used to compare the mRNA level and methylation changes among multiple groups.

A previous study from our team identified two IRF8 SNPs to be associated with BD [12]. We expanded these studies by investigating the IRF8 methylation status in BD patients and whether this affects the gene expression of IRF8. The mRNA expression and methylation status of IRF8 in DCs from active ocular BD subjects was compared with that observed in normal subjects. Our findings revealed that the mRNA expression of IRF8 was notably lower in active ocular BD subjects (*p<0.05) as compared to controls (Figure 1A). To investigate the methylation level of IRF8, a MassARRAY compact MALDI-TOF mass spectrometer was applied to analyze the CpG sites between -441 and -225 from the TSS of the first exon. A Total of 9 CpG sites could be detected. The methylation levels of CpG_1, CpG_11.12.13 and CpG_16 were remarkably increased in DCs of active BD subjects compared to normal subjects (*p<0.05) (Table 1, Figure 1B, 1C, 1D).

**Table 1:** Methylation levels of the IRF8 promotor in active BD patients and normal controls

CpG site	BD Methylation level(%, mean±SD)	NC Methylation level(%, mean±SD)	p value	Postion(from TSS)
CpG_1	6.15 ± 3.13	4.02 ± 2.85	0.0163*	-401
CpG_3	5.38 ± 1.23	5.80 ± 1.93	0.3883	-391
CpG_4, 5, 6	6.93 ± 2.34	7.14 ± 4.04	0.8387	-381, -377, -372
CpG_7,8	22.75 ± 6.39	21.22 ± 4.90	0.3489	-359, -355
CpG_9,10	10.48 ± 2.27	11.52 ± 4.35	0.3258	-349, -346
CpG_11,12,13	9.33 ± 3.85	6.61 ± 3.33	0.0114*	-338, -328, -324
CpG_14	3.85 ± 3.15	3.18 ± 3.32	0.4760	-319
CpG_15	4.94 ± 9.46	4.32 ± 8.37	0.8239	-306
CpG_16	8.28 ± 4.42	5.39 ± 4.14	0.0219*	-299

BD patients (n=20, M:F =19:1); Age (years: mean±SD): 33.40 ± 6.56

Normal controls (n=30, M:F =28:2); Age (years: mean±SD): 34.80 ± 10.07

To study the role of disease activity on IRF8 methylation and IRF8 mRNA expression, we compared a group of active patients with inactive BD individuals. The data showed a higher mRNA expression of IRF8 in the inactive BD patients compared with active subjects (*p<0.05) (Figure 1A). Moreover, the methylation levels of CpG_1, CpG_11.12.13 and CpG_16 were notably decreased in inactive BD subjects compared to the active patients (**p<0.01, ***p<0.001) (Figure 1B, 1C, 1D).

### DAC up-regulated the mRNA expression and down-regulated the methylation level of IRF8 in active BD patients

**Figure 2:**
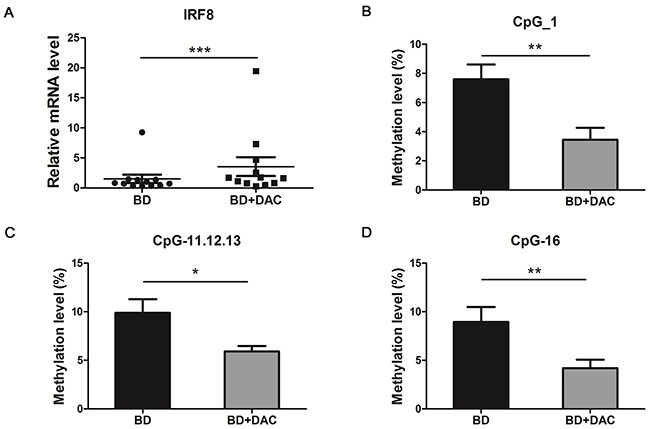
DAC treatment showed a demethylation effect and increased the mRNA expression of IRF8 in DCs from active BD patients DCs from active ocular BD patients were cultured with or without DAC for 6 days, then followed by a stimulation with 100 ng/ml LPS for an additional 24 hours. Then the mRNA expression and the methylation change of IRF8 were evaluate. **(A)** The mRNA expression of IRF8 was measured in DAC-treated and -untreated DCs from active BD patients by real-time PCR (n=12, ***p<0.001). The methylation status of CpG_1 **(B)**, CpG_11.12.13 **(C)** and CpG_16 **(D)** of DAC-treated and -untreated DCs from BD patients was measured with a MALDI-TOF mass spectrometer (n=10, *p<0.05, **p<0.01). The data are shown as mean ± SEM. The paired-t test was used to compare the mRNA level and methylation status between two groups.

To study the demethylation effect of IRF8, a DNA demethylation reagent, DAC, was used to treat DCs for a time period of 6 days, which was then followed by stimulation of the cells with LPS for an additional 24 hours. Both mRNA and DNA were extracted from the DAC-treated and -untreated DCs. Our results showed that DAC treatment restored IRF8 mRNA expression compared to the untreated cells (***p<0.001) (Figure 2A). Correspondingly, the methylation levels were also significantly decreased in the CpG sites which were found to be hypermethylated in our previous set of experiments. The methylation levels of CpG_1, CpG_11.12.13 and CpG_16 of DAC-treated cells were decreased compared with untreated DCs (*p<0.05, **p<0.01) (Figure 2B, 2C, 2D).

### DAC suppressed the expression of co-stimulatory molecules on DCs derived from active ocular BD patients

**Figure 3:**
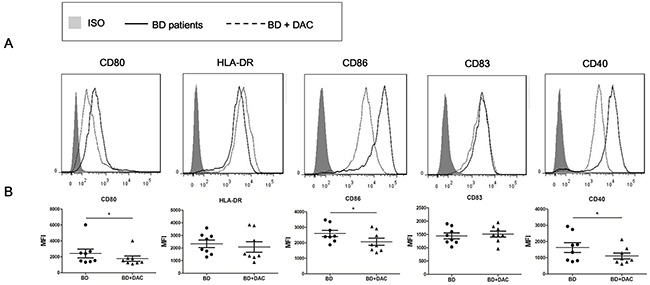
DAC reduces the expression of surface markers of DCs from active ocular BD patients DCs from ocular BD patients were cultured with or without DAC for 6 days, then followed by a stimulation with 100 ng/ml LPS for an additional 24 hours. Cell surface staining of DCs was performed using antibodies against HLA-DR, CD86, CD80, CD83 and CD40, followed by analysis in a flow cytometer. **(A)** Representative histograms with overlays of DAC-treated and -untreated DCs from BD patients. **(B)** The expression of the tested surface markers from DAC-treated and -untreated DCs were analyzed as mean fluorescence intensity (MFI). The data are shown as mean ± SEM. The paired t test was used to compare the MFI between two groups (n=8, *p < 0.05).

The co-stimulatory molecules expressed by DCs are critical for their antigen presentation capability and the further activation of effector Th cells [36]. The effect of demethylation on DC maturation and expression of co-stimulatory molecules and surface molecules, including human leukocyte antigen (HLA)-DR, CD86, CD83, CD80 and CD40 was tested in DAC- treated and -untreated DCs from active ocular BD patients by flow cytometry. We found that the expression of CD86, CD80 and CD40 was notably reduced in DAC treated cells compared with untreated cells (*p<0.05) (Figure 3). However, the expression of HLA-DR or CD83 was not affected by DAC (p>0.05). These findings suggest that demethylation of DCs may have a regulatory effect on DC maturation by diminishing the expression of CD86, CD80 and CD40, which may ultimately inhibit the differentiation of Th cells.

### DAC inhibited the production of DC secreted pro-inflammatory cytokines from ocular BD patients

**Figure 4:**
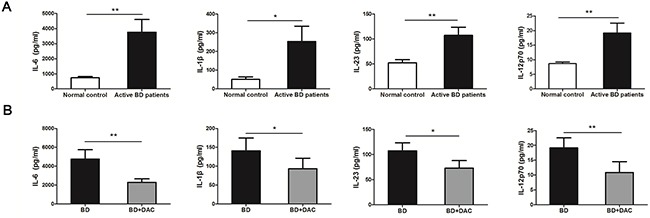
DAC affects the production of inflammatory cytokines by DCs from active BD patients The protein concentrations of IL-6, IL-1β, IL-23 and IL-12p70 in the supernatants of the DCs which were cultured with or without DAC were determined by ELISA. **(A)** The protein concentrations of IL-6, IL-1β, IL-23 and IL-12p70 were significantly increased in BD patients compared with the normal controls (n=12, *p < 0.05,**p < 0.01). The unpaired t-test was performed for statistical analysis. **(B)** The protein concentrations of IL-6, IL-1β, IL-23 and IL-12p70 were remarkably decreased following treatment with DAC (n=12, *p < 0.05,**p < 0.01). The data are shown as mean±SEM. The paired-samples t-test was performed for statistical analysis.

DCs can polarize naïve T cells into different subsets of Th cells by secreting a diversified panel of cytokines [37]. Since earlier studies demonstrated that Th1 and Th17 cells play essential roles in the pathological process of BD [3–5], the effect of demethylation was investigated on the production of Th1/Th17 polarizing cytokines by DCs. The protein expression of Th17-prompting cytokines: IL-6, IL-1β and IL-23 in the ocular BD patients was approximately 5.1-, 5.0- and 2.1-fold higher than observed in normal controls (Figure 4A). Moreover, there was a 2.2-fold increase of IL-12p70 which promotes Th1 responses in BD patients as compared to normal controls (*p < 0.05,** p < 0.01) (Figure 4A). The effect of demethylation was subsequently investigated using DCs from active ocular BD patients. Protein levels of IL-6, IL-1β, IL-23 and IL-12p70 were significantly reduced by approximately 2.1-, 1.5-, 1.5- and 1.8-fold following treatment with DAC, respectively (*p < 0.05,** p < 0.01) (Figure 4B). Fold changes of the protein levels are shown in Supplementary Table 1.

### DAC treatment reduced the frequencies of IFN-γ/IL-17-producing CD4^+^ T cells

**Figure 5:**
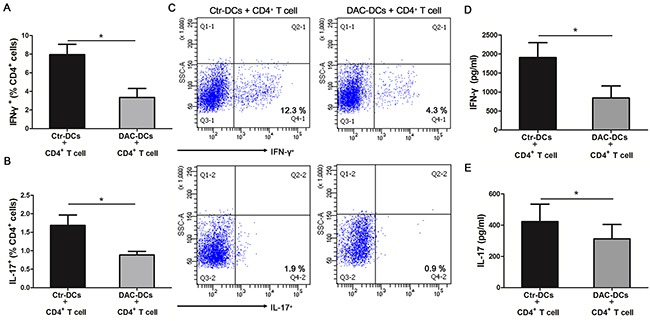
DAC treatment inhibits the Th1 and Th17 responses CD4^+^ T cells from healthy controls were co-cultured with DAC-treated or -untreated DCs (obtained from active ocular BD patients) for 5 days. The frequencies of IFN-γ^+^ **(A)** and IL-17^+^ cells **(B)** in the CD4^+^ T cells co-cultured with DAC-treated or -untreated DCs were determined by flow cytometry (n=7, *p < 0.05). **(C)** The representative results of the The frequencies of IFN-γ^+^ and IL-17^+^ cellsin the CD4^+^ T cells co-cultured with DAC-treated or -untreated DCs. The protein concentrations of IFN-γ **(D)** and IL-17 **(E)** in the cell culture supernatants were detected by ELISA (n=7, *p < 0.05). The data are shown as mean±SEM. DAC-DCs: DAC-treated DCs; Ctr-DCs: control DCs (DAC-untreated DCs). The paired t test was used to compare the protein concentrations of IFN-γ and IL-17 and the frequencies of IFN-γ^+^ and IL-17^+^ cells between the two groups.

DCs play an important role in activating T cells and regulating the differentiation of T helper cell subsets [38, 39]. Therefore, we co-cultured CD4^+^ T cells with DAC-treated or untreated DCs for 5 days to study the demethylation effect of DAC on the Th1/Th17 response. We found that co-culturing with DAC-treated DCs resulted in a significant reduction of IFN-γ^+^ CD4^+^ and IL-17^+^ CD4^+^ cells compared with untreated DCs (control DCs) (*p < 0.05) (Figure 5A, B, C). Furthermore, the protein levels of IFN-γ and IL-17 were remarkably decreased when CD4^+^ T cells had been co-cultured with DAC-treated DCs as compared to co-culture with untreated DCs (*p < 0.05) (Figure 5D, 5E).

## DISCUSSION

In this study, we found that hypermethylation of IRF8 resulted in its gene silencing in DCs of active ocular BD patients. Concomitantly, it was shown that IRF8 methylation was lower and mRNA expression was increased in these DCs, when patients were tested who had entered an inactive phase of the disease. Demethylation treatment with DAC restored the expression of IRF8, inhibited the maturation of DCs and reduced the production of inflammatory cytokines and was associated with a lower Th1/Th17 response in cells obtained from active ocular BD patients. The observation that ocular BD is associated with an aberrant IRF8 status supports the role of this factor in the pathogenesis of this particular uveitis entity. Our results also support previous observations showing that SNPs of IRF8 confer risk to ocular BD [12]. The data are also in agreement with previous studies showing the important role of IRF8 in the control of ocular inflammation in mice with experimental autoimmune uveitis (EAU)[26].

Several studies have shown that IRF8 is associated with several autoimmune diseases, including BD [12, 21, 22]. The role of epigenetic control of IRF8 in autoimmune disease has not been reported yet and our study is among the first to address this issue. An earlier epigenome-wide association study (EWAS) reported DNA methylation changes in the CD4^+^ T cells and monocytes from BD patients [40]. This EWAS found that the aberrant DNA methylation changes were mainly among the genes which regulate cytoskeletal dynamics in the CD4^+^ T cells and monocytes of BD patients but did not identify IRF8 methylation abnormalities [40]. However, we focused on DCs and used a mass spectrometer to detect the methylation changes. Therefore, the different cell types and detection techniques may offer an explanation for the fact that this earlier study did not identify methylation changes in IRF8.

We aimed at studying DCs since these cells are considered to be pivotal in the pathogenesis of BD. Previous studies showed that IRF8 plays a crucial role in regulating the maturation and function of different subsets of DCs [16, 41, 42]. The surface markers expressed on DCs can facilitate TCR signaling and prompt the activation and differentiation of T cells [43]. A higher expression of maturation markers on DCs may reflect disease activity in Behcet's uveitis [44]. Our data showed that demethylation by DAC treatment reduced the expression of the co-stimulatory molecules CD40, CD80, and CD86. The treatment with DAC however did not affect the expression of CD83 or HLA-DR. Our findings indicate that the function of DCs can be regulated by methylation changes, although further studies are needed to clarify whether the observed effects are fully or partially mediated by the methylation changes of IRF8.

A broad spectrum of inflammatory cytokines secreted by DCs is important for the differentiation of different Th cell subsets, including Th1 and Th17 cells. Previous studies showed an increased level of IL-6, IL-1β and IL-23 in DCs from active ocular BD patients [11]. Similarly, our findings revealed that the protein concentration of the Th17-facilitating cytokines, IL-6, IL-1β, IL-23 and the Th1-facilitating cytokine, IL-12p70 were remarkably increased in DCs obtained from active ocular BD subjects. Following *in vitro* demethylation treatment with DAC, the protein expression of these cytokines was markedly decreased.

IRF8 is considered as a novel transcriptional inhibitor for the differentiation of Th17 cell [24]. In addition, a previous study from our group suggested that the suppressing effect of IL-27 on Th17 cell differentiation was associated with an up-regulation of IRF8 [10]. Our demethylation experiments including the co-cultures of DCs and CD4^+^ T cells support the theory that hypermethylation of IRF8 is associated with a decreased IRF8 mediated regulation of DC cell surface marker expression and loss of the control of excessive Th1/Th17 responses.

We realize that our study suffers from some limitations. First of all, it is very difficult to collect a large number of samples from BD patients in a short time period, let alone sampling patients from different categories. Due to the various subgroups, one would need a very large sample size to permit a proper statistical analysis of the data. Thus, in our study, we were only able to investigate the methylation changes in a small cohort of active BD patients and we neither compared the IRF8 methylation status between active ocular BD patients with or without drug treatment nor correlated the methylation changes with the clinico-pathological features of BD patients. Therefore, further studies should be performed to investigate whether immunosuppressive drug treatment used for BD affects DNA methylation and to correlate the methylation changes with the clinico-pathological features. Secondly, due to technical limitations, only a limited region of the promoter could be covered, and not all CpG sites can be detected as in previous studies using the MassARRAY system [45, 46]. Based on these aforementioned limitations, a more comprehensive detection method, such as EWAS is required in future studies to validate the relationship between gene methylation status, disease susceptibility and treatment response. In addition, we used a recently universal-acceptable means to regulate the methylation status by a demethylation reagent, DAC. This method not only targets IRF8, but also affects other genes that might be hypermethylated in BD patients. Demethylation of these genes may partially contribute to the protective effects. Hence, more specific means to regulate the methylation change for a certain gene need to be discovered and applied in the future. Whether IRF8 methylation abnormalities are confined to BD patients with ocular disease is not clear and future studies should be performed to investigate whether BD patients with other disease manifestations show similar abnormalities. It is also not clear whether our findings can be generalized to other uveitis entities, although preliminary findings from our group showed that IRF8 methylation abnormalities can also be found in patients with Vogt-Koyanagi-Harada uveitis (unpublished data). Whether IRF8 methylation status may play a role in diagnosis of ocular BD is not clear. Diagnosis currently relies on the clinical presentation of the patient and as yet we do not see an immediate additional role for IRF8 methylation status as a diagnostic tool, although it might be used as a biomarker to assess disease activity. It is also a bit early to speculate on the role of methylation control of IRF8 in the clinical management of BD, although control of methylation might represent a future target in the treatment of this and other autoimmune diseases.

In conclusion, this study provides the first evidence that hypermethylation of IRF8 in DCs is associated with active ocular BD. The methylation status of IRF8 was closely related to the activity of ocular BD. Demethylation of IRF8 with DAC showed advantageous effects as evidenced by repressing the function of DCs, and ultimately suppressing Th1/Th17 responses. This study emphasizes the potential for epigenetic studies to uncover novel aspects of disease pathogenesis and therefore to identify new targets for the treatment of various autoimmune inflammatory diseases, including BD.

## MATERIALS AND METHODS

### Subjects

Blood samples were collected from 32 Chinese active BD patients (31 males and 1 female, average age: 34.78 ± 7.97 years), 15 inactive BD patients (14 males and 1 female, average age: 37.80 ± 8.16 years) and 47 normal control subjects (37 males and 10 females, average age: 37.02 ± 9.80 years). The enrolled patients were diagnosed in our uveitis center of the First Affiliated Hospital of Chongqing Medical University from December, 2015 to December, 2016. The diagnosis of BD was made in accordance with the diagnostic criteria for Behcet's disease [47]. The basic information of the active and inactive BD subjects are summarized in Supplementary Table 2 and Supplementary Table 3. At the time of sampling, the enrolled BD patients with active ocular inflammation presented the following ocular manifestations: floating cells in the anterior chamber (100%), hypopyon (25%), skin lesions (63%), arthritis (28%), oral ulcers (72%), genital ulcers (41%) and positive skin allergy reactions (9%), respectively. Inactive BD patients included in the study had to show no signs of ocular inflammation for a time period of at least three months. All procedures strictly obeyed the tenets of the Declaration of Helsinki and were approved by the Clinical Ethical Research Committee of Chongqing Medical University. Informed consent was obtained from all the enrolled subjects.

### Cell isolation and culture

DC's derived from monocytes were used for all the assays described in our study. CD14 and CD4 mAb-conjugated magnetic microbeads were used to purify the CD14^+^ monocytes and CD4^+^ T cells from peripheral blood mononuclear cells (PBMCs) of active ocular BD subjects (32 samples), inactive ocular BD patients (15 samples) and the normal controls (47 samples) (purity >90%, Miltenyi Biotec, Germany). The cells were cultured as previously described [11]. Briefly, RPMI 1640 medium which contained 10% fetal bovine serum and 100 U/mL penicillin/streptomycin was used to culture the CD14^+^ monocytes. Monocyte-derived DCs were generated with the stimulation of 100 ng/ml granulocyte macrophage colony stimulating factor (GM-CSF, Acro Biosystems, Newark, USA) and 50 ng/ml human interleukin- 4 (IL-4, Acro Biosystems, Newark, USA) for 3 days. Then, half of the culture medium was refreshed and culture was continued for 3 days.

To investigate the DNA demethylation effect on DC function, DCs were cultured with or without 5-Aza-2′-deoxycytidine (DAC, Sigma-Aldrich, St. Louis, USA) at a concentration of 10 μM for 6 days. Then, the cells were incubated with 100 ng/ml LPS (Sigma-Aldrich, St Louis, USA) for 24 hours to generate mature monocyte-derived DCs. Subsequently, the cells and the supernatants were harvested for further assays. DAC-treated or –untreated mature DCs were co-cultured with CD4^+^ T cells derived from normal subjects (DC: T cell ratio = 1:5) for 5 days. Cell culture supernatants were subsequently collected for ELISA assays of IL-17 and IFN-γ. The cells were then harvested for intracellular detection of IL-17 and IFN-γ by flow cytometry.

### Real-time PCR analysis

Total mRNA was isolated from DCs with TRIzol reagent (Invitrogen, Carlsbad, USA). The PrimeScript RT reagent kit (Takara Biotechnology, Dalian, China) was used to synthesize complementary DNA, and SYBR Premix (Takara Biotechnology, Dalian, China) was used to perform the real-time analysis in a volume of 20 μl with the ABI Prism 7500 system (Applied Biosystems, Foster City, USA) under the conditions described previously: 95°C for 10 minutes, then 40 cycles of 15 seconds under 95°C and 60 seconds at 60°C. Each reaction was run in duplicate. Data were normalized to β-actin. Relative quantification was achieved by the previously described comparative 2^-ΔΔCt^ method [48]. The PCR primer pairs used in this study were shown as follows: β-actin: forward: 5’-ACTGGAACGGTGAAGGTGACAG-3’, reverse: 5’-GGTGGCTTTTAGGATGGCAAG-3’. IRF8: forward: 5’-GAAGACGAGGGTTACGCTGTG-3’, reverse: 5’-TCCTCAGGAACAATTCGGTAA-3’.

### DNA preparation and bisulfite treatment

The QIAamp DNA Blood Kit (Qiagen, Valencia, USA) was used to extract the DNA from DCs in line with the manufacturer's instructions. The EZ DNA Methylation Kit (Zymo Research, Irvine, USA) was used to perform the bisulfite conversion of genomic DNA (800 ng). During the conversion process, the following cycling conditions were repeated for 20 cycles: 95°C for 30 seconds followed by 50°C for 15 minutes.

### Quantitative methylation analysis of IRF8 promotor by Sequenom MassARRAY EpiTYPER System

The selected CpG islands and the primers for MassARRAY analysis were determined as previously described [49]. In brief, DNA sequences of the CpG islands in the IRF8 promoter region were determined by an online database of the University of California, Santa Cruz (available online with open access at http://www.genome.ucsc.edu). Then the primers for the methylation analysis of IRF8 were designed by the EpiDesigner online software (available online with open access at http://www.epidesigner.com) according to the obtained DNA sequences of the CpG islands.

The methylation level of the IRF8 promoter was evaluated by the Sequenom MassARRAY EpiTYPER system. The PCR amplification was initiated at 95°C for 4 minutes, then denaturation at 95°C for 20 seconds for 45 cycles, then annealing under 58°C for 30 seconds, followed by 72°C for 1 minute with a final incubation at 72°C for 3 minutes. The primer of IRF8 covered from -441 bp to -225 bp up-stream from the transcription initiation site (TSS) of the first exon. Methylation primer of IRF8 was as shown: forward: 5’-aggaagagagGGGTAGTTAGTTTTTGGTTGTGGAT-3’; reverse: 5’-cagtaatacgactcactatagggagaaggctTACAAAAAAACTTTCCCAAAAATTC-3’. The shrimp alkaline phosphatase (SAP) and MassCLEAVE reaction were performed according to the manufacturer's instructions. The end-products were desalted and dispensed to a SpectroCHIP using a MassARRAY™ Nanodispenser (Sequenom, Inc., San Diego, USA). Then a matrix assisted laser desorption ionization time-of-flight (MALDI-TOF) mass spectrometer (Sequenom, Inc., San Diego, USA) was used to acquire the spectra. The methylation level of IRF8 promoter was analyzed with the MassARRAY EpiTYPER (Sequenom, Inc., San Diego, USA) and the results were shown as percentages of methylation of each CpG site. The results were normalized by the methylation level of the test standard within the methylation analysis kit (Sequenom, Inc., San Diego, USA).

### Flow cytometry

To detect the effect of DAC on the DC surface markers, DAC-treated and -untreated DCs were stained by anti-human CD86-APC, CD80-PE, HLA-DR-PE/Cy5, CD83-PE and CD40-PerCP-Cy5.5 (all antibodies were from BioLegend, San Diego, USA) at 4°C for 30 minutes. The expression of cell surface markers was detected by flow cytometry (FACSAira, BD Biosciences, Franklin Lakes, USA) and analyzed as the median fluorescence intensity (MFI). The histograms with overlays were made by FlowJo software (Treestar, Inc., San Carlos, USA).

To investigate whether DAC affects the function of DCs in relation to its effect on differentiating T cells into Th1/Th17 subsets, CD4^+^ cells were co-cultured with DAC-treated or -untreated DCs for 5 days as previously described [11]. Then 100 ng/ml PMA (Sigma-Aldrich, St. Louis, USA) and 1 μg/ml ionomycin (Sigma-Aldrich, St. Louis, USA) were added to the cells at 37°C for 1 hour and subsequently incubated for an additional 4 hours with 10 μg/ml brefeldin A (Sigma-Aldrich, St. Louis, USA). Anti- IFN-γ-FITC and IL-17A-PE antibodies (both from eBioscience, San Diego, USA) were used to perform the intracellular staining for 30 minutes at 4°C. The frequencies of CD4^+^ IFN-γ^+^ and CD4^+^ IL-17A^+^ cells were detected with the FACSAira flow cytometer (BD Biosciences, Franklin Lakes, USA).

### Enzyme-linked immunosorbent assay (ELISA)

The protein levels of IL-6, IL-1β, IFN-γ and IL-17 were detected by Duoset ELISA kits (R&D Systems, Minneapolis, USA). The expression of IL-12p70 and IL-23 were evaluated by the IL-23 ELISA Ready-SET-GO kit and the IL-12(p70) high sensitivity ELISA kit, respectively (both from eBioscience, San Diego, CA, USA) based on the manufacturer's instructions.

### Statistical analysis

Data are shown as mean±SEM. SPSS 22.0 software (SPSS Inc, Chicago, Illinois, USA) and GraphPad Prism 5 software (GraphPad Software, Inc., San Diego, CA, USA) were used for the statistical analysis. The statistical significance of differences between two comparisons was determined by independent-sample t-test or paired-samples t-test. One way-ANOVA followed by Bonferroni correction was used to analyze the statistical differences among multiple groups. A p-value less than 0.05 was considered to be significantly different.

## SUPPLEMENTARY MATERIALS TABLES



## Abbreviations

APCs: antigen presenting cells
BD: Behcet's disease
DCs: dendritic cells
DAC: 5-Aza-2′-deoxycytidine
EAU: experimental autoimmune uveitis
EWAS: epigenome-wide association studies
GM-CSF: granulocyte macrophage colony stimulating factor
HLA: human leukocyte antigen
IRF8: interferon regulatory factor 8
IL-4: interleukin-4
MALDI-TOF: matrix assisted laser desorption ionization time-of-flight
MFI: median fluorescence intensity
PBMCs: peripheral blood mononuclear cells
SNP: single nucleotide polymorphism
SAP: shrimp alkaline phosphatase
Th: T helper
TSS: transcription initiation site
