# Supplementary material for: Ocular Behcet’s disease is associated with aberrant methylation of interferon regulatory factor 8 (IRF8) in monocyte-derived dendritic cells
Source: Oncotarget. 2017 Apr 19;8(31):51277–87. doi: 10.18632/oncotarget.17235 (PMC5584248; doi:10.18632/oncotarget.17235)
Supplement: Supplementary file 1 [file oncotarget-08-51277-s001.pdf]

Ocular Behcet's disease is associated with aberrant methylation of interferon regulatory factor 8 (IRF8) in monocyte-derived dendritic cells

SUPPLEMENTARY TABLES

Supplementary Table 1: Protein level fold changes of inflammatory cytokines

Group	Cytokines	Protein fold changes	p value	Sample size	Statistical methods
Nornal controls vs. BD patients	IL-6	5.1	0.0017	12	Unpaired t-test
	IL-1β	5.0	0.0227	12	Unpaired t-test
	IL-23	2.1	0.0045	12	Unpaired t-test
	IL-12p70	2.2	0.0036	12	Unpaired t-test
BD vs. BD+DAC	IL-6	2.1	0.0056	12	paired-samples t-test
	IL-1β	1.5	0.0274	12	paired-samples t-test
	IL-23	1.5	0.0249	12	paired-samples t-test
	IL-12p70	1.8	0.0039	12	paired-samples t-test

Supplementary Table 2: Basic information of the active ocular BD patients enrolled in the study

Patient number	Age	Gender	Medications
Case 1	31	F	No drugs
Case 2	34	M	PDN: 15 mg, qd + CsA: 50 mg, bid
Case 3	33	M	PDN: 15 mg, qd + CsA: 75 mg, bid
Case 4	27	M	PDN: 15 mg, qd + CsA: 75 mg, bid + CTX: 50 mg, qd
Case 5	33	M	PDN: 20 mg, qd + CsA: 75 mg, qd + CTX: 50 mg, qd
Case 6	30	M	PDN: 20 mg, qd + CsA: 75 mg, qd + CTX: 50 mg, qd
Case 7	32	M	PDN: 5 mg, qod for 4 months
Case 8	32	M	PDN: 20 mg, qd + CsA: 50 mg, bid
Case 9	38	M	No drugs
Case 10	56	M	PDN: 20 mg, qd + CsA: 125 mg/day
Case 11	20	M	No drugs
Case 12	28	M	PDN: 10 mg, qd
Case 13	39	M	No drugs for 4 months
Case 14	26	M	PDN: 20 mg, qd + CsA: 50 mg, qd + CTX: 50 mg, qod
Case 15	40	M	No drugs for 2 months
Case 16	45	M	No drugs for 6 months
Case 17	27	M	PDN: 20 mg, qd + CsA: 125 mg/day
Case 18	38	M	PDN: 20 mg, qd + CTX: 50 mg, qd
Case 19	20	M	PDN: 20 mg, qd + CTX: 50 mg, qd
Case 20	46	M	No drugs
Case 21	34	M	PDN: 20 mg, qd + CTX: 50 mg, qd
Case 22	39	M	PDN: 20 mg, qd + CsA: 25 mg, bid + CTX: 50 mg, q3d
Case 23	28	M	PDN: 20 mg, qd + CsA: 50 mg, qd
Case 24	37	M	No drugs
Case 25	27	M	PDN: 20 mg, qd + CsA: 75 mg, qd
Case 26	45	M	PDN: 15 mg, qd + CsA: 125 mg/day
Case 27	34	M	PDN: 20 mg, qd + CsA: 75mg, qd
Case 28	29	M	PDN: 10 mg, qd + CsA: 50 mg, bid
Case 29	39	M	PDN: 20 mg, qd + CsA: 50 mg, bid
Case 30	45	M	PDN: 15 mg, qd + CsA: 125 mg/day
Case 31	44	M	PDN: 10 mg, qd
Case 32	37	M	PDN: 20 mg, qd + CsA: 50 mg, qd

PDN: Prednisone; CsA: cyclosporin a; CTX: cyclophosphamide

Supplementary Table 3: Basic information of the inactive ocular BD patients enrolled in the study

Patient number	Age	Gender	Medications
Case 1	42	F	PDN: 15 mg, qd + IFN: $3*10^7$ IU, qd
Case 2	34	M	PDN: 15 mg, qd + CsA: 75 mg, qd
Case 3	38	M	PDN: 5 mg, qd + CsA: 50 mg, bid
Case 4	48	M	PDN: 10 mg, qd + CsA: 50 mg, bid
Case 5	45	M	PDN: 5 mg, qd + CsA: 50 mg, bid
Case 6	43	M	PDN: 15 mg, qd + CsA: 50 mg, bid
Case 7	29	M	PDN: 15 mg, qd + CsA: 125 mg/day
Case 8	21	M	PDN: 10 mg, qd + IFN: $3*10^7$ IU, qod
Case 9	40	M	PDN: 15 mg, qd + CsA: 50 mg, qd
Case 10	37	M	PDN: 20 mg, qd + CsA: 125 mg/day
Case 11	48	M	IFN: $3*10^7$ IU, qw
Case 12	31	M	IFN: $3*10^7$ IU, q3d
Case 13	47	M	PDN: 5 mg, qd
Case 14	27	M	PDN: 5 mg, qd + CsA: 75 mg, qd
Case 15	37	M	PDN: 20 mg, qd + CsA: 75 mg, qd

PDN: Prednisone; CsA: cyclosporin a; IFN: Interferon α
